# Dystrophic Epidermolysis Bullosa

**DOI:** 10.31729/jnma.3791

**Published:** 2018-10-31

**Authors:** Randhir Sagar Yadav, Amar Jayswal, Shumneva Shrestha, Sanjay Kumar Gupta, Upama Paudel

**Affiliations:** 1 Maharajgunj Medical Campus, Institute of Medicine, Tribhuvan University, Kathmandu, Nepal; 2 Department of General Practice and Emergency Medicine, Maharajgunj Medical Campus, Institute of Medicine, Tribhuvan University, Kathmandu, Nepal; 3 Department of Dermatology, Maharajgunj Medical Campus, Institute of Medicine, Tribhuvan University, Kathmandu, Nepal

**Keywords:** *blister*, *dystrophic epidermolysis bullosa*, *epidermolysis bullosa*, *knee disarticulation*, *surgery*

## Abstract

Epidermolysis bullosa is a rare inherited blistering disease with an incidence of 8–10 per million live births. Dystrophic epidermolysis bullosa is a type of epidermolysis bullosa caused by mutation in type VII collagen, COL7A1. There are 14 subtypes of dystrophic epidermolysis bullosa and 400 mutations of COL7A1. Electron microscopy is the gold standard diagnostic test but expensive. Immunofluorescence study is a suitable diagnostic alternative. Trauma prevention along with supportive care is the mainstay of therapy. Squamous cell carcinoma develops at an early age in epidermolysis bullosa than other patients, particularly in recessive dystrophic epidermolysis bullosa subtypes. Regular follow-up is imperative in detecting and preventing complications. Gene therapy, cell therapy and bone marrow transplantation are the emerging novel therapeutic innovations. Preventing possible skin and mucosal injury in patients requiring surgery should be worked on. Here, we present a case of dystrophic epidermolysis bullosa in a 26-year-old male.

## INTRODUCTION

Epidermolysis bullosa (EB) is an inherited, rare genetic blistering disorder precipitated by mechanical stress.^1^ Based on level of tissue separation, there are four major classes of EB, namely simplex, junctional, dystrophic and Kindler syndrome.^2^ Dystrophic EB (DEB) results due to mutation in the type VII collagen, COL7A1.^2^ EB has an estimated prevalence of 8–10 per million live births where DEB accounts for 2–6 per million live births. The prevalence is similar in both sexes.^3^ Even though definitive diagnosis is concluded from specific genetic mutation, meticulously taken history and watchful physical examination can help in making the initial diagnosis.^3, 4^

CASE REPORT

**Figure 1A-B:**
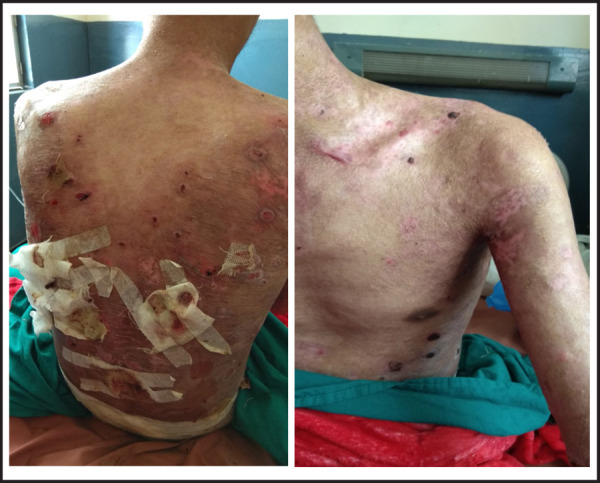
Multiple vesicles and bullae with erosions and hemorrhagic crusts measuring approximately 0.5x0.5 cm to 3x5 cm over back. Ruptured blisters are covered with dressing. Hypopigmented areas seen at the sites where previous lesions have healed.; Crusts over few lesions on left shoulder, arm and abdomen with hypopigmentation over areas of previously healed lesions.

**Figure 2.**
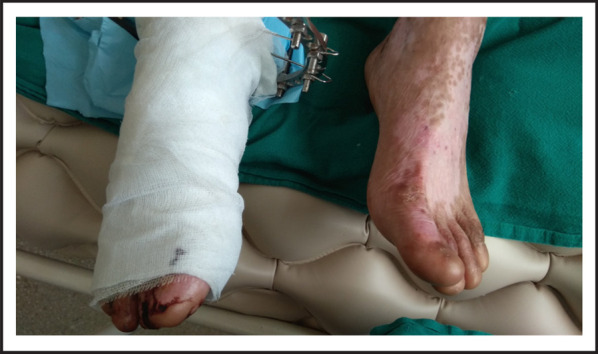
Right leg with Illizarov prior to below knee disarticulation. His grade IIIC fracture of middle one-third of both bones of right leg was managed with Illizarov and K-wire. Bilateral feet without nails since birth. Few crusted lesions following blister rupture along with hypopigmented areas at previously healed lesions.

A 26-year-old male was referred for further management of absent distal pulsation of right leg following road traffic accident which was unsalvageable. There were multiple vesicles and bullae with erosions and hemorrhagic crusts present (Figure 1). There were hypopigmented and atrophic areas of previously healed lesions (Figure 1, 2). Nails in his bilateral hands and feet were absent (Figure 2).

A diagnosis of DEB was made. Direct i immunofluorescence was advised for definitive subtype classification which was deferred by patient for financial reasons. He was managed symptomatically with Condy's wash followed by dressing with mupirocin ointment and paraffin gauge. He was discharged after his lesions improved with proper counselling.

There were multiple vesicles over buccal mucosa of approximately 0.5x0.5 cm but no dental involvement. Pre-operative laboratory evaluation revealed reactive thrombocytosis and anemia. Rest of the investigations were within reference range. Two pints of packed cell volume were transfused maintaining adequate hydration. Oral Febuxostat and Hydroxycarbamide were given. Hemoglobin raised from 8.2gm/dl to 11mg/dl and platelet decreased from 12,24,000///l to 8,00,000/ /yl. Right knee disarticulation was done under general anesthesia with laryngeal mask airway. Post-operative period was uneventful except for appearance of few new bullae.

Similar blisters erupted since birth following traumas. Skin biopsy seven years back at a different center made a histopathological diagnosis of epidermolysis bullosa. He developed progressive dysphagia and odynophagia six years ago. Barium swallow study revealed persistent smooth narrowing of entire cervical, upper and mid-thoracic part of esophagus which was managed conservatively. Since then his dysphagia was static. There were similar but relatively smaller and less numerous vesicles in a son and a daughter of his paternal aunt. There was no consanguineous marriage in family.

## DISCUSSION

DEB has been classified depending on the extent of type VII collagen expression, clinical manifestations and pattern of inheritance. The latest classification has divided DEB into 14 subtypes.^1^ Likewise, there are 400 mutations in COL7A1 gene.^3^ “DDEB (Dominant DEB), generalized”, “RDEB (Recessive DEB), generalized severe” and “RDEB, generalized intermediate” are three most common variants of DEB.^3^ “DDEB, generalized” expresses reduced amount of collagen VII and has a good prognosis.^1, 5^ Blisters are mild, limited to area of trauma. Oral mucosa and teeth involvements are rare.^3^

“RDEB, generalized severe”, previously known as Hallopeau-Siemens has absence or markedly reduced amount of type VII collagen and thus its most severe subtype of DEB.^1, 5^ It has various extracutaneous manifestations involving kidney, heart, gastrointestinal and genitourinary tract.^3^ Oral cavity, esophagus, eyes and anal canal manifestations are seen due to mucus membrane involvement.^1^

“RDEB, generalized intermediate” shows clinical manifestations of “RDEB, generalized severe” but with less severe blistering as it has type VII collagen expression but in reduced amount.^5^ It was previously named as non Hallopeau-Siemens type or “RDEB, generalized other”.^1^ It has better prognosis than “RDEB, generalized severe”.^3^ Moreover, squamous cell carcinoma (SCC) can develop in any EB patient and is seen at an early age than non-EB cases. RDEB subtype cases have high chances of developing SCC. SCC in RDEB develops at younger age, has shorter duration than DDEB. Therefore, all EB cases, RDEB subtypes in particular necessitate regular follow-up.^6^

Following careful clinical and inheritance pattern evaluation, skin biopsy from a recent blister is advised to evaluate tissue separation depth.^3, 4^ History of consanguinity is also important.^7^ Since light microscopy can only differentiate between intraepidermal and subepidermal splits, immunofluorescence and electron microscopy are recommended.^1, 3^ Electron microscopy is expensive so immunofluorescence study is a valuable diagnostic alternative.^3, 4^ Mutational analysis gives most accurate subclassification which has role in prognostication, genetic counseling as well as prenatal diagnosis.^5^ Acral and oral involvement along with esophageal manifestations were evidenced in our case as well, however there were no dental lesions. Diagnosis in our case was based on clinical and inheritance pattern evaluation and skin biopsy.

There is no specific treatment of ED where symptomatic management remains mainstay of treatment.^3^ New blisters and trauma prevention, appropriate wound care with dressing is vital in patient care.^8^ Nutritional support along with early detection and prevention of complications like skin infection, scar formation, skin cancers and extracutaneous complications are important.^7, 8^ Furthermore, multifactorial approach and multidisciplinary involvement with psychosocial support is needed.^5, 7, 8^ Blisters are recommended to be punctured to avoid its dissemination. Skin is left in the original place to act as a natural barrier. Lesions are covered with nonstick dressings like paraffin gauge.^3^ Skin should be lubricated with Vaseline or bland ointments.^8^ Daily dressing is suggested. Compressive and adhesive dressings must not be used as it can produce new blisters.^3^ Topical antibiotics and antimicrobial dressings are ought to be used prudently in DEB cases.^3^ Our case was also managed by multidisciplinary team with dressing, topical antibiotic as well as stump, skin and supportive care. Skin graft, bone marrow transplant, allogeneic fibroblast injection, recombinant protein therapy, gene therapy and stem cell therapy are advent of future advanced treatment.^3^

Monitoring, transport and positioning are important issues for EB patients necessitating surgery,^9^ which was well considered in our case. Providing anesthesia is an additional challenges,^9^ so laryngeal mask airway was used. Amputations are usually avoidable in EB patients.^6^ Mobility in EB patients is an essential aspect.^7^ Success of fitting prosthesis despite persistent wound at stump has been reported,^10^ which was discussed with our patient to enhance his mobility and quality of life.
